# Implementation and Efficacy of Total Wellness: A Community-Based Cancer Prevention and Lifestyle Intervention Program

**DOI:** 10.1007/s13187-025-02589-z

**Published:** 2025-03-29

**Authors:** Devon C. Riegel, Jamila L. Kwarteng, Laura Pinsoneault, Sandra Contreras, Erica Wasserman, Alexis Visotcky, Ana Manriquez Prado, Oscar Villarreal Espinosa, Patricia Sheean, Margaret Tovar, Kathleen Jensik, Regina M. Vidaver, Melinda R. Stolley

**Affiliations:** 1 Medical College of Wisconsin, Cancer Center, Milwaukee, WI USA; 2 Medical College of Wisconsin, Institute for Health and Equity, Milwaukee, WI USA; 3 Evaluation Plus LLC, Milwaukee, WI USA; 4 Milwaukee Public Schools, Milwaukee Recreation, Milwaukee, WI USA; 5 Department of Statistics, Medical College of Wisconsin, Milwaukee, WI USA; 6 Department of Applied Health Sciences, Loyola University Chicago, Chicago, IL USA; 7 Wisconsin Department of Health Services, Division of Public Health, Madison, WI USA

**Keywords:** Cancer, Primary prevention, Health disparities, African Americans, Hispanic Americans, Community-based

## Abstract

Cancer is the second leading cause of death in Wisconsin, with higher rates in Black/African American (Black) and Hispanic/Latino (Hispanic) populations. In partnership with Milwaukee Recreation (MKE Rec), Total Wellness (TW) was created to provide lifestyle and cancer prevention programming to local communities. Program feasibility, potential efficacy, and sustainability were evaluated. TW is a 16-week program integrated into MKE Rec and implemented over two 8-week sessions led by instructors trained in lifestyle change and cancer prevention. TW was evaluated at the individual and systems level. Class registrants were invited to participate in the evaluation, which included surveys and a physical assessment. Sixty participants consented to the program evaluation: 58.3% self-identified as Black and 7.3% as Hispanic. Survey results indicated significant improvements in cancer knowledge, self-reported diet, and physical activity. Physical assessment showed significant improvements in weight, body mass index, blood pressure, and fitness. System-wise, three MKE Rec instructors were trained, and classes operated out of two sites, serving a predominantly Black neighborhood and a large Hispanic population. English-taught sessions of TW showed successful integration into MKE Rec programming throughout 2 years. Spanish-language only classes were attempted but did not meet minimum enrollment to proceed. TW showed success in improving individual cancer awareness and healthy lifestyle changes. TW was also integrated into MKE Rec and showed sustained success in reaching Black communities with limited reach in Hispanic communities. This program demonstrates the potential for a community health intervention program to address cancer disparities.

**Supplementary Information:**

The online version contains supplementary material available at 10.1007/s13187-025-02589-z.

## Introduction

Cancer is the second leading cause of death in the USA [1]. Minoritized groups, including Black/African American (Black) and Hispanic/Latino (Hispanic) populations, are disproportionately affected by cancer compared to non-Hispanic whites (white). Black men have the highest mortality rate from all cancers; Black women have a 40% higher chance of dying from breast cancer than white women; and, prior to 2020, cancer was the leading cause of death among Hispanics [1]. Social determinants of health (SDH), including low access to healthy lifestyle resources, drive these disparities [2].

Many cancers can be prevented by modifying risk behaviors [3]. The American Cancer Society (ACS) developed guidelines to inform and promote cancer risk-reducing behaviors [4]; however, awareness and adherence are low for most Americans. Additionally, although evidence-based interventions (EBIs) are accessible to public health practitioners for the design and implementation of cancer prevention programs [5, 6], the rate of implementation remains low due to challenges such as inadequate funding or lack of skilled personnel [7–9].

Total Wellness (TW) is an evidence- and community-based cancer prevention and lifestyle intervention program. This study examined the feasibility and potential efficacy of the program in producing improvements in cancer knowledge, social support, health behaviors, and fitness. We also explored program sustainability within this real-world implementation.

## Methods

### Total Wellness Program Design

Total Wellness was developed and implemented through a partnership between the Medical College of Wisconsin Cancer Center (MCWCC) and the Milwaukee Recreation System (MKE Rec). TW was adapted from Moving Forward (MF) [10, 11], which focused on behavioral and lifestyle strategies for weight loss among Black breast cancer survivors. The adaptation process, which engaged a community advisory board to integrate cancer prevention and screening information, was presented at a national conference [12] and will be detailed in a subsequent paper.

Total Wellness is a 16-week program promoting adherence to the ACS nutrition and physical activity guidelines [4]. The TW program is grounded in social cognitive theory (SCT) [13] and the socio-ecological model (SEM) [14]. Based on these models, TW was developed to (1) enhance participant knowledge to make health-related lifestyle changes, (2) increase social support to facilitate lifestyle change, and (3) improve access to community resources for cancer prevention and overall wellness.

Led by specially trained MKE Rec instructors, TW was implemented over two 8-week sessions (TW 1.0, 2.0) and met twice weekly. Class 1 included an interactive cancer and lifestyle education session guided by a program booklet targeting weekly topics (see Supplemental Table A) and addressing knowledge, attitudes, and behavioral strategies to accomplish and maintain lifestyle changes. Weeks 6 and 10 featured plant-based cooking demonstrations. The 30-min learning time was followed by a 60-min exercise class with flexibility, strength, and cardio training. Class 2 was a 60-min exercise class only.

Total Wellness remains available as a standing MKE Rec class each season at two sites, one serving a predominately Black community and the other serving a largely Hispanic community. Classes serving the Hispanic community were offered in English and Spanish-speaking formats. Interested individuals register via phone, in-person, or on the MKE Rec class registration website. The program costs $8 for each 8-week session.

MKE Rec recruited three certified fitness instructors with nutrition knowledge and interest in cancer prevention to participate in TW instructor training; the lead instructor was a registered dietitian nutritionist (RDN). Training was led by two behavioral scientists and a nutrition scientist with expertise in lifestyle, nutrition, cancer, and cancer disparities. Training content covered basic cancer biology, risk factors, behavior change, screening/early detection, and a summary of common treatments for the four most commonly diagnosed cancers. Bilingual English/Spanish speaking instructors were recruited to teach the Spanish-language classes.

### Program Marketing

The MCWCC and MKE Rec invested in program marketing using several streams: (1) MKE Rec direct advertising to current MKE Rec clients and posting program information on their website; (2) MCWCC community advertising and posting on their website; and (3) community partner and physician/healthcare clinic advertising.

### Program Outcomes Evaluation

*Overview*.

The TW evaluation used a pre-post study design to assess individual and systems-level outcomes with measures based on ease of participant completion and feasibility of implementation within MKE Rec sites. Individual outcomes were assessed following TW 1.0 (8 weeks) and TW 2.0 (16 weeks). Class participants were invited to complete the evaluation once they registered for the class; evaluation completion was not required for enrollment. The systems level evaluation explored feasibility and sustainability using the RE-AIM framework (reach, effectiveness, adoption, implementation, maintenance) [15].

*Ethics*.

The program evaluation procedures were reviewed and approved for ethical treatment of human subjects by the Medical College of Wisconsin Institutional Review/Ethics Board.

*Recruitment*.

Following TW enrollment through MKE Rec, MKE Rec staff inquired whether class enrollees were interested in the evaluation and obtained their permission to be contacted by study staff. Subsequently, research staff reached out to explain the evaluation and screened for eligibility.

*Eligibility for the Evaluation*.

Inclusion criteria: (1) enrolled in the MKE Rec TW class; (2) at least 18 years of age.

Exclusion criteria: (1) planning to move out of state within study duration; (2) answered “Yes” to 3/3: currently adhering to ACS guideline for (a) fruits/vegetables (> 5 a day), (b) physical activity (> 150 min moderate intensity or 75 min vigorous intensity), and (c) strength training (> 2x/week); (3) pregnant, less than 3 months post-partum, or planned to become pregnant within the next year; (4) answered “yes” to 1/7 initial questions on the Physical Activity and Exercise Readiness Questionnaire + (PARQ +) [16] and did not receive physician approval to participate in the evaluation.

### Individual Outcome Measures

Participants completed surveys via a secure RedCap link and physical assessments at the MKE Rec site for the TW programs prior to the first day of TW 1.0 class, at the end of TW 1.0, and at the end of TW 2.0. Surveys were available in English and Spanish and included (1) demographics and health history, (2) cancer knowledge [17], (3) perceived safety and access to healthy lifestyle resources [18], (4) social support [19], (5) Patient Reported Outcomes Measurement Information System-29 (PROMIS) v2.0 [20], (6) Godin Leisure Time Exercise Questionnaire (GLTE) [21], and (7) Dietary Screener Questionnaire (DSQ) [22]. See supplement for additional descriptions.

Physical assessment measures included the following: (1) height/weight/body impedance analysis (BIA), (2) waist circumference, (3) blood pressure (BP), (4) sit-to-stand, (5) hand grip strength, and (6) 6-min walk. See supplement for additional descriptions.

### System Outcome Measures

System-level evaluation data points were grounded in the RE-AIM framework [15]: Reach, number of participants enrolled in class, number of teachers trained (in English and/or Spanish language); Efficacy, intervention impact on outcomes; Adoption, number of settings and instructors that implemented the program; Implementation, interventionist’s fidelity to TW implementation and clients’ use of intervention strategies (as reflected by behavior change); and Maintenance, TW integration into MKE Rec programming. Additionally, we define feasibility as sufficient registration (≥ 5 individuals/session) to TW classes over the 2-year study period, and satisfaction using a questionnaire at 16 weeks.

### Statistical Analysis

Outcomes are presented with means and standard deviations at baseline, 8, and 16 weeks. A non-parametric signed rank test was used to compare outcomes over time. Analysis was done using SAS Version 9.4 (SAS Institute Inc., Cary, NC).

## Results

### Demographics

Over 2 years, 126 individuals registered for the program and 60 consented to participate in the evaluation. Of those consented, 58 participants completed baseline surveys and physical assessments, 53 completed these at 8 weeks and 33 at 16 weeks. Participant demographics are summarized in Table 1. Mean (standard deviation [SD]) age was 53.50 (13.81), and the majority self-identified as female (84.2%). A total of 58.3% self-identified as Black, 38.3% as White, and 7.3% self-identified as Hispanic. A total of 32.7% reported being current or former tobacco users.

**Table 1:** Participant characteristics

Variables	*N* = 60 (%)
**Age**	
Mean ± SD	53.50 ± 13.81
Missing	2
**Race**^a^	
Black/African American	35 (58.3)
White	23 (38.3)
American Indian/AN	2 (3.3)
Asian	2 (3.3)
**Ethnicity**	
Non-Hispanic or Latino	51 (92.7)
Hispanic or Latino	4 (7.3)
Missing	5 (8.3)
**Education**	
High school graduate or GED	2 (3.5)
Associate’s degree or 2-year certificate	7 (12.3)
Some college	9 (15.8)
College graduate	18 (31.6)
Graduate or professional degree	21 (36.8)
Missing	3 (5)
**Employment**	
Employed for pay full-time	30 (51.7)
Employed for pay part-time	4 (6.9)
Out of work	1 (1.7)
Retired	22 (37.9)
Other	1 (1.7)
Missing	2 (3.3)
**Marital status**	
Single, never married	17 (29.8)
Married or living with a partner	25 (43.9)
Widowed	6 (10.5)
Separated	1 (1.8)
Divorced	8 (14.0)
Missing	3 (5)
**Income**	
Less than $20,000	1 (1.7)
$20,000–$39,999	5 (8.6)
$40,000–$59,999	14 (24.1)
$60,000–$79,999	9 (15.5)
$80,000 or more	17 (29.3)
Prefer not to answer	12 (20.7)
Missing	2 (3.3)
**Overall health rating**	
Fair	9 (15.5)
Good	35 (60.3)
Very good	10 (17.2)
Excellent	4 (6.9)
Missing	2 (3.3)
**Type of health insurance**	
Private insurance	41 (68.3)
Medicare	14 (23.3)
Medicaid	4 (6.7)
Other	2 (3.3)
**Health history**^b^	
Diabetes	8 (13.8)
High blood pressure	24 (42.1)
High cholesterol	23 (39.7)
Heart disease	3 (5.2)
Emphysema or COPD	2 (3.4)
Asthma	10 (17.5)
Arthritis or back problems	22 (37.9)
Thyroid disorders	3 (5.2)
Blood disorders	4 (6.9)
Sleep apnea	8 (14.0)
Cancer	4 (7.1)
**Tobacco use**	
None	39 (67.2)
Current	3 (5.2)
Former tobacco use	16 (27.6)
Missing	2

^a^Participants could indicate more than one race

^b^Percentages based on number of respondents with 2–4 missing responses for all diagnoses

### Individual Outcomes

Table 2 provides results for cancer knowledge, perceived access, social support, and PROMIS. Cancer knowledge was relatively high at baseline with improvements seen at 8 and 16 weeks (*P* = 0.006, 0.007). Perceived Neighborhood Safety also improved at 8 weeks (*P* = 0.05). PROMIS T scores at baseline were mostly in keeping with population averages (T = 50), but participants reported higher burden with sleep disturbance (52.9 ± 3.1). In contrast, participants’ fatigue (45.4 ± 8.3) and depression scores (46.0 ± 6.8) indicated less burden than the general population, and their ability to participate in social roles was higher (55.1 ± 8.4). No significant differences were observed for social support or PROMIS subscales.

**Table 2:** Cancer knowledge, perceived access, social support, and PROMIS outcomes at 8 and 16 weeks

Outcome	Baseline Mean ± SD	8-week Mean ± SD	16-week Mean ± SD	*P* value 8-week - Baseline	*P* value 16-week - Baseline
	*N* = 58	*N* = 53	*N* = 33		
**Cancer knowledge**					
% correct	87.3 ± 9.4	90.1 ± 9.9	91.9 ± 8.5	.006	.007
**Perceived access**					
Perceived access for exercise	6.3 ± 1.8	5.8 ± 1.7	6.1 ± 1.8	.22	.77
Perceived access for healthy eating	7.9 ± 2.6	8.0 ± 3.2	7.6 ± 2.7	.72	.90
Perceived neighborhood safety	12.4 ± 1.4	12.0 ± 1.4	12.2 ± 1.5	.05	.69
**Social support for:**					
Eating habits encouragement family	18.8 ± 12.6	14.9 ± 9.4	17.4 ± 11.0	.26	.93
Eating habits discouragement family	18.4 ± 12.3	15.1 ± 10.4	16.0 ± 10.9	.51	1.0
Eating habits encouragement friends	14.1 ± 10.7	13.5 ± 10.2	15.7 ± 11.4	.75	.74
Eating habits discouragement friends	13.8 ± 11.3	13.7 ± 11.8	14.5 ± 11.1	.86	.79
Exercise participation family	30.1 ± 22.7	29.3 ± 20.4	31.5 ± 19.4	.64	.14
Exercise participation friends	26.1 ± 19.8	30.8 ± 21.5	30.8 ± 20.2	.19	.78
**PROMIS measures (T-score)**					
Physical function	52.2 ± 6.4	52.7 ± 6.5	52.8 ± 6.4	.31	.76
Depression	46.0 ± 6.8	46.1 ± 7.1	47.5 ± 6.9	.98	.36
Anxiety	49.9 ± 8.0	50.5 ± 8.0	49.9 ± 8.9	.47	.62
Fatigue	45.4 ± 8.3	46.6 ± 8.9	45.8 ± 7.4	.14	.76
Sleep disturbance	52.9 ± 3.1	52.4 ± 4.6	53.3 ± 3.4	.18	.81
Pain interference	28.9 ± 8.0	48.4 ± 8.0	50.1 ± 8.2	.55	.49
Ability to participate in social roles and activities	55.1 ± 8.4	56.1 ± 7.9	55.6 ± 7.6	.52	.36

Health behavior results are shown in Table 3. Mean (SD) GLTE scores at baseline were 25.4 (22.1), indicative of being sufficiently active; average screen time daily was 4 h. Fruit and vegetable consumption was moderate at baseline; added sugars was high. Following TW, GLTE scores increased (8 weeks* P* = 0.0014; 16 weeks *P* = 0.0034) with more participants characterized as sufficiently active (8 weeks *P* = 0.0012; 16 weeks *P* = 0.0013). Vegetable consumption increased at 8 weeks (*P* = 0.042) but this was not maintained at 16 weeks (*P* = 0.086). Added sugar consumption decreased (8 weeks *P* = 0.018; 16 weeks *P* = 0.021). The frequency of red and processed meat intake trended downward during TW participation. Specifically, the frequency of red meat consumption decreased from baseline to 16 weeks (*P* = 0.036), while the frequency of processed meat consumption decreased from baseline to 8 weeks (*P* = 0.022). Whole grain and fruit intakes did not change significantly.

**Table 3:** Godin Leisure Time Exercise Scale, Dietary Screener Questionnaire, anthropometrics, cardiovascular vitals, and fitness measures at 8 and 16 weeks

Outcome	Baseline Mean ± SD/%	8-week Mean ± SD/%	16-week Mean ± SD/%	*P* value 8-week - Baseline	*P* value 16-week - Baseline
	*N* = 58	*N* = 53	*N* = 33		
**Godin leisure time exercise scale**					
Total	25.4 ± 22.1	35.8 ± 22.2	38.6 ± 21.5	.0014	.0034
**Godin categories**					
Insufficient/moderate (≤ 23)	60%	31.4%	30%		
Sufficient (≥ 24)	40%	68.6%	70%		
Change in Godin category				.0012	.0013
**Daily hours screen time**	4.4 ± 2.0	3.7 ± 1.8	3.7 ± 1.9	.01	.017
**Dietary Screener Questionnaire**					
Whole grains (oz)	.6 ± .2	.6 ± .3	.5 ± .1	.54	.13
Fruits (cups)	1.0 ± .4	1.0 ± .4	1.0 ± .4	.22	.81
Vegetables (cups)	1.5 ± .04	1.5 ± .5	1.6 ± .5	.042	.086
Total added sugars (tsp)	14.9 ± 5.4	13.5 ± 3.7	13.0 ± 3.0	.018	.014
**Anthropometrics**					
Weight (pounds)	199.6 ± 47.1	193.4 ± 49.1	190.2 ± 52.9	.52	.0014
BMI (kg/m^2^)	33.0 ± 6.9	32.6 ± 7.2	32.3 ± 7.4	.50	.0016
Waist circumference (cm)	107.7 ± 14.2	105.5 ± 16.6	104.3 ± 17.7	.29	.099
**Cardiovascular vitals**					
Systolic BP (mm/hg)	126.1 ± 17.2	123.0 ± 17.5	119.7 ± 15.1	.028	.012
Diastolic BP (mm/hg)	77.8 ± 12.1	74.9 ± 11.1	74.8 ± 11.7	.011	.034
Heart rate (bpm)	77.8 ± 12.5	77.8 ± 14.4	76.3 ± 14.2	.61	.40
**Fitness measures**					
Non dominant hand grip (kgs)	27.6 ± 7.7	29.1 ± 8.5	29.7 ± 8.8	< .001	.057
Dominant hand grip (kgs)	29.0 ± 7.5	30.3 ± 8.3	30.7 ± 8.2	.016	.065
Sit to stand # reps/30 secs	12.4 ± 4.9	15.2 ± 5.7	17.4 ± 6.2	< .001	< .001
6 min walk (meters)	486.1 ± 75.2	519.8 ± 91.5	536.9 ± 95.5	< .001	< .001

Physical assessment results are summarized in Table 3. BMI at baseline was 33.0 (SD = 6.9). Average systolic BP (SBP) was elevated (126.1 ± 17.2); diastolic BP (DBP) was within normal range (77.8 ± 12.5). Weight and BMI decreased at 16 weeks (weight *P* = 0.0014; BMI *P* = 0.0016). Average SBP and DBP decreased at 8 and 16 weeks (SBP *P* = 0.028, 0.012; DBP *P* = 0.011, 0.034). No significant differences were observed in waist circumference or average heart rate.

Sit-to-stand repetitions increased at 8 and 16 weeks (*P* < 0.001, < 0.001). Dominant hand average hand grip strength increased at 8 weeks (*P* = 0.016) and non-dominant hand average grip strength increased at 8 and 16 weeks (*P* < 0.001, 0.057). Distance on the 6MWT increased at 8 and 16 weeks (*P* < 0.001, < 0.001).

### System Outcomes

Total Wellness showed moderate success in its integration into MKE Rec programming, as outlined in the RE-AIM framework.

*Reach*.

A total of 126 participants enrolled in all TW class sites over two sites in two years; 60 consented to participate in the program evaluation (47.6%). Only a subset of TW 1.0 participants (N = 45) continued to TW 2.0. While some classes were filled to capacity (N = 15) per MKE Rec’s COVID-19 policy, 4 of 24 offered classes failed to reach minimum enrollment (N = 5). Spanish-only sections were offered twice; neither met minimum enrollment.

*Efficacy*.

Participant-level data reflect improvements in knowledge, behavior, and health outcomes.

*Adoption*.

Total Wellness was adopted at two sites with no new sites added.

*Implementation*.

Fidelity data reflect TW was implemented as intended: 16 classes over two 8-week sessions; led by trained MKE Rec instructors; 2 × weekly classes offering cancer and lifestyle information based on pre-established topics (Table 2); and 60-min supervised exercise classes with resistance and cardio training.

*Maintenance*.

TW 1.0 and TW 2.0 were listed in the MKE Rec course catalog every season for the last 2 years and continues to be available. Program evaluation participants reported high program satisfaction. Furthermore, following participation in TW 1.0 and 2.0, the first class of TW program registrants requested a TW 3.0 so TW participants could continue to exercise together as a group. Approximately 20 participants continued to participate via this outlet.

## Discussion

Our goal was to examine the feasibility, potential efficacy, and program sustainability of the TW program. Study results highlight the value of the TW program. Individual-level data support potential efficacy with significant improvements in health behaviors (diet, physical activity) and also in markers of physical health, including BMI, blood pressure, and increased strength and endurance. Systems level results support program feasibility and potential sustainability, demonstrated by program adoption at two sites, relatively consistent enrollment over 2 years, instructor retention, maintenance of integration into MKE Rec programing, and participant satisfaction. Notably, despite offering two seasons of the Spanish-language classes, these never reached sufficient enrollment for implementation.

Total Wellness was developed using a community-engaged and iterative approach to ensure community and cultural relevance. Previous research shows behavioral interventions employing culturally adapted strategies impact participant outcomes [23]. While program satisfaction is high and outcomes are positive, program enrollment and retention have wavered. Program awareness is an on-going challenge; MKE Rec offers thousands of classes and residents tend to return to familiar classes. Despite diversified advertising efforts, the Spanish-language TW sessions failed to achieve sufficient enrollment. This may be explained by the fact that TW was the first Spanish-language program offered by MKE Rec, and despite marketing efforts, awareness remained low. Furthermore, Milwaukee has community centers better known to Hispanic communities providing comprehensive services in education, health, and community development. Future endeavors will consider collaborations between MKE Rec and these organizations to offer TW.

Previous literature indicates that community-based cancer prevention programs are effective; however, they are often limited by short duration and lack of sustainability within existing community resources [24, 25]. Public recreation systems such as MKE Rec serve as excellent examples of well-established community partners whose support can facilitate longevity of cancer prevention programming. The ultimate goal is to maintain the current sites and to further disseminate TW to other MKE Rec sites for broader reach.

An important goal of TW is to create a community of support for cancer prevention. Community support in TW was expanded by creating TW 3.0, and the consistent engagement with this class demonstrates TW’s impact on social support systems. However, it is important to highlight that participants overall reported higher than population average ability to participate in social roles at baseline, and there was no significant difference in social support results post-intervention. This may be explained in part by the MKE Rec community itself; several TW participants had previously participated in other MKE Rec programming. Furthermore, individuals who did participate may have been actively involved in other community organizations (e.g., churches) where TW was being advertised. Notably, subjective responses collected from the post-16-week satisfaction questionnaire did indicate that several participants appreciated the “support” from the class as well as the “class structure” to facilitate long-term change.

This study is not without limitations. The lack of randomization and the opt-in nature of the evaluation contribute to risk of sample bias. Sample bias is further reflected in the attrition in TW 2.0 registration and evaluation participation at 16 weeks. However, this also raises a question about program duration and whether an 8-week program would suffice, given the robust results observed following TW 1.0. A larger implementation study will need to consider this with a more robust study design such as a cluster randomized trial or stepped-wedge design. Additionally, behavioral measures were all self-reported and thus vulnerable to over- or under-reporting. However, the inclusion of objective measures of fitness and strength contribute to study rigor. Finally, participant results indicated relatively high baseline cancer knowledge as well as sufficient levels of physical activity at baseline via Godin score average ≥ 24 [21]. This suggests a need for improved outreach methods to engage populations with lower baseline cancer knowledge and in need of physical activity lifestyle interventions; all of which could contribute to even greater community cancer prevention impact.

Based on individual-level and system-level data, we conclude that implementing cancer prevention programming into a public recreations system is feasible, can influence cancer-related behavioral risk factors, and holds promise for sustainability. Future directions include modifications to outreach strategies to better engage Hispanic communities. Efforts to reach populations with lower cancer knowledge and greater lifestyle intervention needs will also be initiated.

## Supplementary Information

Below is the link to the electronic supplementary material.

Supplementary file1 (DOCX 24.2 KB)

## Data Availability

Deidentified data may be provided upon request and at the investigators' discretion.
