# Supplementary material for: Implementation and Efficacy of Total Wellness: A Community-Based Cancer Prevention and Lifestyle Intervention Program
Source: J Cancer Educ. 2025 Mar 29;40(6):886–93. doi: 10.1007/s13187-025-02589-z (PMC12717134; doi:10.1007/s13187-025-02589-z)
Supplement: Supplementary file 1 — Supplementary file1 (DOCX 24.2 KB) [file 13187_2025_2589_MOESM1_ESM.docx]

**Supplement**

Individual Outcome Measures: Surveys

1. Demographics and health history: name, address, birthdate, marital status, education level attained, occupational status, annual income, insurance status, health history.
2. Cancer Knowledge: An adapted version of the Cancer, Clear and Simple Cancer Education Curriculum assessment^17^ containing 15 true/false items related to cancer and preventive lifestyles.
3. Perceived Access to Healthy Eating and Exercise: Respondents rate their level of agreement to statements on safe access to physical activity and healthy eating resources. Internal consistency coefficients range from 0.78 to 0.94.^18^
4. Social support: respondents rate how often friends and family do or say things related to respondents’ efforts to change dietary or exercise habits. Internal consistency coefficients range from 0.74 to 0.81.^19^
5. Quality of Life: The Patient Reported Outcomes Measurement Information System (PROMIS) provides a standardized metric for measuring physical, mental, and social health.^20^ Scores use a common metric (T-score with a mean of 50 and standard deviation of 10) and have been normed to the US population. Higher scores represent more of that domain.
6. Pain: The PROMIS-29 v2.0^20^ asks participants to rate their daily experience of pain on a scale of 0-10, 0 = no pain at all, and 10 = worst pain they have experienced.
7. Godin Leisure Time Exercise Questionnaire (GTLE): evaluates weekly participation in strenuous, moderate, and/or mild physical activity for at least 15 minutes.^21^ We added a question to assess frequency of strength training. Scoring ≤23 indicates insufficiently to moderately active, and scoring ≥24 indicates sufficiently active.
8. Dietary Screener Questionnaire (DSQ): a 26-item dietary assessment tool available in Spanish and English asking participants about the frequency of consumption in the past month of fruits and vegetables, dairy/calcium, added sugars, whole grains/fiber, red meat, and processed meat.^22^ Algorithms to calculate serving sizes are not validated for red meat and processed meat. Therefore, these data are reported as frequency.

Individual Outcome Measures: Physical Assessments

1. Height/Weight/Body Impedance Analysis (BIA): measured and used to calculate body mass index (BMI). Body Impedance Analysis is a non-invasive measure of body composition and was measured using an FDA-approved device (Tanita 430U).
2. Waist circumference: measured using a Gulick measuring tape at the umbilicus.
3. Blood Pressure (BP): measured using an automated blood pressure machine and a standardized protocol.
4. Sit-to-stand: measures leg strength and endurance; participants keep their hands crossed over their chest with hands placed on opposite shoulders and perform as many sit-to-stand motions as possible in a 30-second period.
5. Hand grip strength (Takei 5401 Hand Grip Digital Dynamometer): participants have 3 trials per hand, alternating between hands to ensure 1-minute rest periods for each hand between trials.
6. 6-minute walk: measures aerobic capacity and endurance, participants are instructed to walk as far as possible for 6 minutes along a 30-meter track. Distances are rounded down to the nearest 5-meter marking.

| **Supplemental Table A** Total Wellness Weekly Topics | | | |
| --- | --- | --- | --- |
| Week | Lifestyle Topic | Cancer Topic | |
| 1 | Introduction to Total Wellness & Goal Setting | What is Cancer? | |
| 2 | Plant-Based Eating | Cancer Risk Factors | |
| 3 | Getting on Track | Cancer & Obesity | |
| 4 | Managing Stress | Cancer & Stress | |
| 5 | Tools to Help You Succeed | Physical Activity & Cancer | |
| 6 | Good Food, Good Budget Cooking Demo | Alcohol & Cancer | |
| 7 | Work in Your Daily Workout | Breast Cancer & Screening | |
| 8 | Self-Review & Goal Setting | Colorectal Cancer & Screening | |
| 9 | What helps, what gets in the way (individual, family, community) | Prostate Cancer & Screening |  |
| 10 | Plant-Based Cooking Demo: replacing red/processed meats | Tobacco, Lung Cancer & Screening | |
| 11 | Cues to Action (individual and community) | Cervical Cancer & Screening | |
| 12 | Mindful Eating | Human Papilloma Virus (HPV) & Vaccine | |
| 13 | Let’s Get Moving | Sunscreen, Skin Cancer & Screening | |
| 14 | Family First | Breastfeeding & Breast Cancer | |
| 15 | Maintaining Changes & Activating Community Part I | Sleep & Your Health | |
| 16 | Activating Community Part II & Celebration |  | |
